# Five main phases of landscape degradation revealed by a dynamic mesoscale model analysing the splitting, shrinking, and disappearing of habitat patches

**DOI:** 10.1038/s41598-019-47497-7

**Published:** 2019-07-31

**Authors:** Ádám Kun, Beáta Oborny, Ulf Dieckmann

**Affiliations:** 1 Evolution and Ecology Program, International Institute of Applied System Analysis, Schlossplatz 1, A-2361 Laxenburg, Austria; 2 Parmenides Center for the Conceptual Foundations of Science, Parmenides Foundation, Kirchplatz 1, D-82049 Pullach, Germany; 3 Department of Plant Systematics, Ecology and Theoretical Biology, Loránd Eötvös University, Pázmány Péter sétány 1/C, H-1117 Budapest, Hungary; 4 MTA-ELTE Theoretical Biology and Evolutionary Ecology Research Group, Pázmány Péter sétány 1/C, H-1117 Budapest, Hungary; 5 Evolutionary Systems Research Group, MTA Centre for Ecological Research, Klebelsberg Kuno u. 3., H-8237 Tihany, Hungary; 6 GINOP Sustainable Ecosystems Group, MTA Centre for Ecological Research, Klebelsberg Kuno u. 3., H-8237 Tihany, Hungary; 7 Department of Evolutionary Studies of Biosystems, The Graduate University for Advanced Studies (Sokendai), Hayama, Kanagawa 240-0193 Japan

**Keywords:** Computational models, Ecological modelling, Population dynamics, Theoretical ecology

## Abstract

The ecological consequences of habitat loss and fragmentation have been intensively studied on a broad, landscape-wide scale, but have less been investigated on the finer scale of individual habitat patches, especially when considering dynamic turnovers in the habitability of sites. We study changes to individual patches from the perspective of the inhabitant organisms requiring a minimum area for survival. With patches given by contiguous assemblages of discrete habitat sites, the removal of a single site necessarily causes one of the following three elementary local events in the affected patch: splitting into two or more pieces, shrinkage without splitting, or complete disappearance. We investigate the probabilities of these events and the effective size of the habitat removed by them from the population’s living area as the habitat landscape gradually transitions from pristine to totally destroyed. On this basis, we report the following findings. First, we distinguish four transitions delimiting five main phases of landscape degradation: (1) when there is only a little habitat loss, the most frequent event is the shrinkage of the spanning patch; (2) with more habitat loss, splitting becomes significant; (3) splitting peaks; (4) the remaining patches shrink; and (5) finally, they gradually disappear. Second, organisms that require large patches are especially sensitive to phase 3. This phase emerges at a value of habitat loss that is well above the percolation threshold. Third, the effective habitat loss caused by the removal of a single habitat site can be several times higher than the actual habitat loss. For organisms requiring only small patches, this amplification of losses is highest during phase 4 of the landscape degradation, whereas for organisms requiring large patches, it peaks during phase 3.

## Introduction

In today’s world, habitat fragmentation is a mostly anthropogenic process that threatens whole ecosystems^1–5^. Mitigating the consequences of, or altogether avoiding, habitat fragmentation is one of the main focuses of conservation ecology^6–9^. Theoretical landscape ecology can aid such conservation efforts^10^ by supplying reliable measures of habitat fragmentation^11,12^ and by analysing models of population and metapopulations to predict the consequences of habitat fragmentation^13–18^. In particular, neutral landscape models (NLMs)^19,20^ have been introduced to provide a standard to which real landscape patterns can be compared.

**Figure 1:**
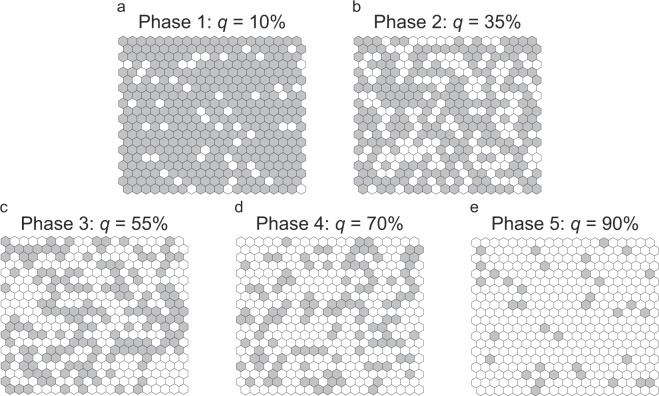
Levels of habitat loss and their differential consequences for habitat patches. Habitat sites (grey hexagons) and non-habitat sites (white hexagons) are distributed randomly on a hexagonal lattice. The shown levels of habitat loss *q* illustrate the five main phases of habitat degradation identified in this study (Table 1): the percentages indicated above the panels are mid-interval values characteristic of Phases 1–5. For example, *q* = 10% means that 10% of habitat cover is lost and that 90% of habitat cover remains, which is the mid-interval value characteristic of Phase 1 (Table 1). The upper two panels show connected landscapes (Phases 1–2, where the level of habitat loss is below the percolation threshold, *q* < *q*_c_), while the lower three panels show fragmented landscapes (Phases 3–5, where *q* > *q*_c_). The pictures illustrate relatively small lattices (20 × 20 sites). In contrast, all numerical investigations in this study were carried out on larger lattices (100 × 100 sites), and a landscape’s property of being connected or fragmented is defined in the theoretical limit of infinite lattice size.

The simplest NLM is the percolation map^20^ (Fig. 1). In a percolation map, habitat sites and non-habitat sites are randomly distributed according to the fraction *p* of habitat sites, with *q* = 1 − *p* denoting the fraction of non-habitat sites. Such models are being extensively studied by physicists, within the field of percolation theory^21^. Percolation theory shows that in an infinite landscape of randomly distributed habitat and non-habitat sites (physicists sometimes refer to these as open and closed sites, respectively), an infinite cluster (also known as giant component or spanning patch) of habitat sites exists above a critical fraction *p*_c_ of habitat sites, called the percolation threshold, whereas below that fraction no such cluster exists. Consequently, the probability that a randomly chosen habitat site belongs to the infinite cluster is zero below the percolation threshold and positive above it. The threshold-like transition from connected to fragmented landscapes observed in percolation maps is instilling caution regarding the dangers of such drastic transitions occurring also in real landscapes^20,22,23^.

In recent years, NLMs have proliferated, becoming more and more sophisticated (for a review of landscape-generating algorithms, see^24^). For example, percolation maps have been generalized to incorporate more than two landscape-cover types^13,25^ or a gradient across which *p* varies from one end of the landscape to the other^26–28^. Also, as real landscapes generally show aggregated patterns, aggregation has been introduced to NLMs. For example, Gustafson and Parker^29^ randomly placed rectilinear clumps with random edge lengths onto a grid to create an aggregated pattern. Hiebler^30^ proposed an iterative method to produce landscapes with given pair-correlation probabilities (see also^31^). In hierarchical random landscapes^32^, the probability of assigning a patch to one of two landscape-cover types is first applied at larger spatial scales before being applied to the remaining habitat at smaller spatial scales, an approach that can be generalized to multiple landscape-cover types^33,34^. Realistically looking mosaic landscapes can also be generated by tessellation methods^35,36^, by fractal methods^37–40^, or by modifying/distorting existing landscape patterns to modify their spatial characteristics^41^. Many of the aforementioned methods are implemented in a readily available software package^42^. Finally, NLMs have been extended to three dimensions, to model soil layers or forest canopies^43^.

The spatially explicit modelling of ecological processes^44,45^ has become the focus of many ecological studies^46^. In the past three decades, an increasing number of population models have incorporated environmental heterogeneity^15,25,30,31,39,47–58^: the resultant body of literature has underscored how NLMs can aid the development of increasingly insightful and realistic landscape models. Compared to NLMs, landscape patterns or patch structures used in experiments are usually simpler, such as a chessboard mosaic of resource-rich and resource-poor patches^59–61^ or patterns based on percolation maps^62–64^. To apply the insights derived from such simple experimental settings to more complex landscapes, suitable models are required, as experimental settings with more complex landscapes^65,66^ are rare.

An important development in NLM approaches has been the incorporation of temporal fluctuations into models^23,49,52,53,67–73^ and experiments^74^. In particular, Hagen-Zanker and Lajoie^69^ have proposed a neutral model of landscape change, introducing a novel method for generating a landscape with multiple landscape-cover types and aggregation. Dynamic neutral landscape models (DNLMs), in general, eschew pre-defined spatial and temporal structures, to study the emergence of these structures from interactions among the components.

The DNLM we employ in the present study can arguably be considered as a minimalistic DNLM, because the process for changing the landscape is very simple and does not induce any spatial or temporal correlations among the habitability of the sites. Consequently, the habitat sites, initially being distributed randomly (as in NLMs), persist being free from any spatial or temporal correlations.

Even this simple representation of a dynamic landscape of habitable sites produces non-trivial emergent phenomena. Many interesting features have already been revealed before, especially by percolation theory elucidating the size distribution of habitable patches (percolation clusters) and the global connectivity of such patches (see^23^ for a review). In general, percolation theory has focused on global features, applying at the scale of the whole landscape, while assuming a static landscape. Here, we extend this view to dynamic landscapes and to the finer scale of individual patches.

This mesoscale is intermediate between the global scale (of the landscape as a whole) and the local scale (of individual habitat sites). From the perspective of an organism inhabiting the landscape, this intermediate scale of habitable patches is particularly important. When non-habitat sites are difficult to traverse for such an organism, it may be confined to a single patch for its whole life (concrete examples concerning plants are discussed by^57^). Thus, any event changing the size of the patch the organism is inhabiting is crucial for the organism.

**Figure 2:**
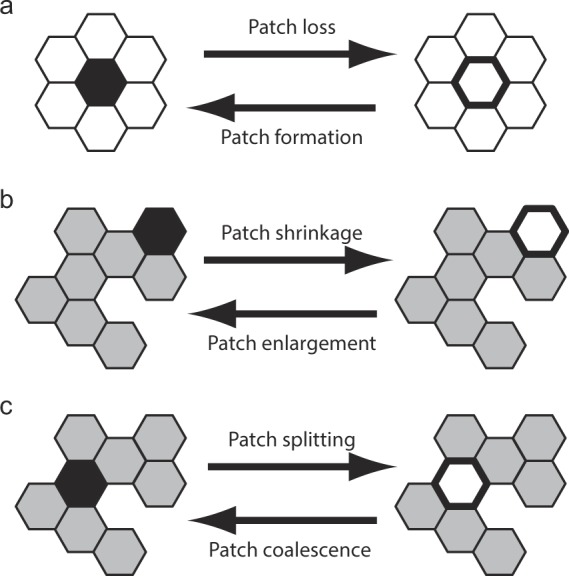
Examples of elementary local events affecting habitat patches on a hexagonal lattice. Non-habitat sites are represented by white hexagons, while habitat sites are represented by grey hexagons. In each row, the hexagon with the thick black outline or black filling, respectively, is the focal one that changes from right to left or from left to right. The following descriptions refer to the elementary events occurring from left to right. (**a**) The loss of the focal habitat site causes the loss of the entire habitat patch, which consisted of a single site only. (**b**) The loss of the focal habitat site from the perimeter of a habitat patch decreases the size of that patch. (**c**) The loss of the focal habitat site from a bottleneck of a habitat patch results in the splitting of that patch; in the shown example, the patch splits into two smaller patches (splitting into three smaller patches is possible for other patch configurations).

Important elementary events on the patch scale have been reviewed before, by Forman^75^ (pp. 407), Jaeger^76^, Akçakaya^77^, and Didham *et al*.^78^. Here we adopt this event-based view and develop it through a quantitative analysis of the events. Specifically, we characterize the relative event frequencies in dependence on the sizes of the patches that are involved. Following Akçakaya^77^, we consider changes in both directions: habitat loss and habitat gain. Accordingly, we systematically distinguish and analyse six elementary local events that can affect a patch (Fig. 2):

**Patch formation** is the appearance of a habitat site within a non-habitat area,

**Patch loss** is the event occurring when a habitat patch ceases to exist by the removal of its last site,

**Patch enlargement** is the addition of a habitat site to an existing habitat patch,

**Patch shrinkage** is the removal of a habitat site from an existing habitat patch,

**Patch coalescence** is the joining of two habitat patches that were previously isolated, and

**Patch splitting** is the division of a habitat patch into two or more isolated ones.

The event-based description of landscape dynamics enabled by the definition of these elementary events offers a general framework for studying patch dynamics in changing landscapes. Here, we apply this general framework to a simple DNLM, a random map with random local fluctuations. We also assume that the landscape-level habitat pattern is in a steady state when habitat loss occurs. We investigate the consequences of a single local habitat loss for the affected patch and analogously study the consequences of a single local habitat gain (see the corresponding loss/gain events in Fig. 2).

Our analysis underscores that landscape fragmentation is not only a problem resulting from the loss of connections between habitat patches, but also a problem related to a minimum feasible patch size. The habitat patches arising from dynamical habitat fragmentation may be smaller than the minimal area needed for sustaining a viable local population of the focal organism. Therefore, by removing a single unit of habitat, a potentially much larger area may be lost from the organism’s perspective. We estimate the magnitude of this kind of loss, and predict the level *q* of habitat loss at which it is the most perilous.

## Methods

The probabilities of the elementary events, as defined above, cannot be obtained analytically for a given landscape, as this would require the full enumeration of all patches together with their sizes and shapes. The number of possible patch shapes or configurations grows exponentially with patch size^79–82^. An enumeration of patch configurations has been achieved only up to a patch size of 45 sites^83^, later pushing this limit to 56 sites^84^. This poses a problem, since percolation maps without habitat patches larger than 56 sites are either very small or have very low values of *p*.

Therefore, we use Monte Carlo simulations to obtain the functions describing how the probabilities of the elementary events depend on the habitat cover *p*, by recording all events taking place on a percolation map with random fluctuations in site habitability. For this purpose, we use lattices consisting of *N* = 100 × 100 = 10,000 sites with periodic boundaries (i.e., the lattice is wrapped around a torus). Initially, *pN* randomly chosen sites are habitat sites and *qN* sites are non-habitat sites.

In our model, we assume a constant proportion *q* = 1 − *p* of non-habitat sites. Thus, when a randomly chosen habitat site is turned into a non-habitat site, at the same time a randomly chosen non-habitat site is turned into a habitat site. For each value of *q*, we record 6 · 10^6^ elementary events. The record of an event consists of the type of the event and of the sizes of all the patches affected by the event. Since the total proportion of habitat sites does not change, the probabilities of patch loss and patch formation are equal, the probabilities of patch enlargement and patch shrinkage are equal, and the probabilities of patch splitting and patch coalescence are equal (see the pairs of opposing processes in Fig. 2). For this reason, it suffices to show results for the three elementary local events of patch loss, patch shrinkage, and patch splitting.

The probability of patch loss can be calculated analytically, assuming an infinite lattice. This enables us to compare our numerical results to an analytical baseline. Patch loss occurs when the local configuration matches the case shown in Fig. 2a. The probability of encountering such a local configuration at a random site of a hexagonal lattice (also referred to as a honeycomb lattice or equilateral triangular lattice) is *p*(1 − *p*)^6^, whereas it is *p*(1 − *p*)^4^ on a square lattice (considering the von Neumann neighbourhood, i.e., the four nearest neighbours). This example illustrates that the probabilities of elementary events are sensitive to the geometry of the considered lattice. Also the percolation threshold, which is analytically known for simple lattice geometries^21^, varies with the geometry of the considered lattice: *p*_c_ = 0.5 (*q*_c_ = 0.5) for the hexagonal lattice and *p*_c_ = 0.592746 (*q*_c_ = 0.407254) for the square lattice with a four-site neighbourhood. The threshold value is exact for the hexagonal lattice and is numerically estimated for the square lattice. In line with these differences, we must expect that the geometry of the lattice affects patch dynamics and the probabilities of the elementary events. For this reason, we include both the hexagonal lattice and the square lattice in our investigations.

For the hexagonal lattice, our numerical investigations are based on values of *p* ranging from 0.05 to 0.95 with 0.05 increments and from 0.45 to 0.55 with 0.01 increments; in addition, the values 0.57, 0.63, and 0.67 were also considered. For the square lattice, our numerical investigations are based on values of *p* ranging from 0.05 to 0.95 with 0.05 increments and from 0.45 to 0.80 with 0.01 increments.

## Results

In this section, we identify three kinds of landscape transitions that are important in addition to the traditionally recognized percolation transition. The resultant four transitions naturally subdivide the process of habitat loss into five phases. Finally, we show how the effective habitat loss experienced by organisms having different requirements for minimal patch sizes increases with the actual habitat loss.

### Three impacts of habitat loss

**Figure 3:**
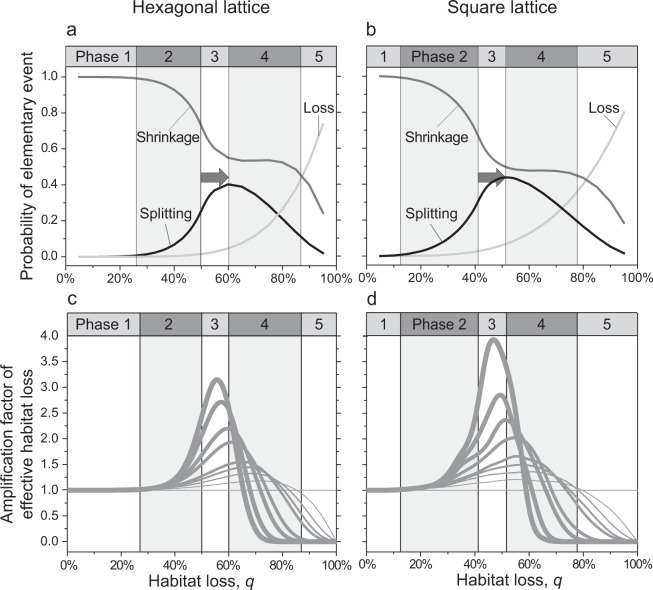
Probabilities of elementary local events affecting habitat patches on a (**a**) hexagonal lattice and (**b**) square lattice, together with the corresponding amplification factors of effective habitat loss on a (**c**) hexagonal lattice and (**d**) square lattice. The curves in panels a and b show how the probabilities of patch loss (light-grey curves), patch shrinkage (dark-grey curves), and patch splitting (black curves) vary with the level of habitat loss *q*. The vertical lines indicate the thresholds separating the five phases of habitat loss identified in this study, which are indicated by alternating white and light-grey backgrounds. Note that for both lattice geometries, the probability of patch splitting peaks at levels of habitat loss that are larger than the percolation threshold; the differences are highlighted by the grey arrows. In panels c and d, the vertical axis shows the amplification factor of effective habitat loss, i.e., the average number of habitat sites lost from a population’s living area caused by the loss of one habitat site, when habitat patches of at least *m* habitat sites are required to sustain a viable local population. The horizontal line shows the amplification factor of effective habitat loss for *m* = 1, in which case the removal of a single habitat site always causes the loss of no more and no less than one habitat site from the population’s living area. The thickness of the curves increases with increasing *m* from 1 up to 9 sites. Notice that the maximum amplification factors of effective habitat loss are observed for levels of habitat loss *q* in excess of the percolation threshold situated at the transition between Phases 2 and 3. Also note that for high levels of habitat loss, very few habitat patches remain viable, which means that most removals of habitat sites affect habitat patches in which the population is not viable, resulting in the amplification factor of effective habitat loss converging to zero.

The functions describing how the probabilities of patch loss, patch shrinkage, and patch splitting change with the level of habitat loss *q* turn out to have similar shapes for the hexagonal lattice and the square lattice (Fig. 3a,b). The numerically obtained probabilities of patch loss perfectly match the aforementioned analytical predictions, increasing monotonically as the level of habitat loss *q* is raised. The probability of patch splitting reaches its maximum at a value of *q* that is higher than the percolation threshold (*q* > *q*_c_). For the hexagonal lattice, patch splitting is most probable when ca. 60% of the habitat is lost (Fig. 3a), while for the square lattice this peak occurs at ca. 50% of habitat loss (Fig. 3b). From there up to ca. 80% of habitat loss, the probability of patch shrinkage remains relatively constant, independent of the two considered lattice geometries (Fig. 3a,b).

### Four transitions caused by habitat loss

We observe the probabilities of the elementary events during the degradation from a pristine landscape (*q* = 0) to the disappearance of all habitat (*q* = 1). Based on the results shown in Fig. 3, we propose to divide the process of habitat loss into five phases, each corresponding to a certain interval of habitat loss. The boundaries between adjacent phases are determined by four major transitions in landscape structure and dynamics occurring during the course of landscape degradation:

**Transition 1** (occurring at a level of habitat loss of ca. 20%). Appearance of isolated patches and a very slowly increasing incidence of patch splitting. This transition can be defined to occur at the level of habitat loss *q* at which the probability of patch shrinkage falls below 99%.

**Transition 2** (occurring at a level of habitat loss of ca. 45%). Transition from a fully connected to a fragmented landscape. This transition happens at the percolation threshold, which is defined in, and well known from, the literature on percolation processes^21^.

**Transition 3** (occurring at a level of habitat loss of ca. 55%). Switch from an increasing to a decreasing probability of patch splitting. This transition is thus defined to occur at the level of habitat loss *q* at which the patch-splitting probability peaks.

**Transition 4** (occurring at a level of habitat loss of ca. 85%). Sudden decline in the probability of patch shrinkage. This transition happens when the probability of patch loss starts to dominate, and we can define it to occur at the habitat loss *q* at which the probability of patch loss surpasses the probabilities of patch shrinkage and patch splitting.

The four values of habitat loss *q* listed above are just rough indications, mentioned here only for the sake of approximate orientation. Naturally, the exact values depend on the details of the model, and in particular on the considered lattice geometry (Table 1).

**Table 1:** Five phases of habitat loss.

Phase of landscape degradation	Estimated habitat loss on a hexagonal lattice (%)^a^	Estimated habitat loss on a square lattice (%)^b^	Approximate indicative range of habitat loss (%)^c^	Amplification factor of effective habitat loss^d^	Landscape connectivity^e^	Response of patch splitting to further habitat loss^a,b^	Response of patch shrinkage to further habitat loss^a,b^	Response of patch loss to further habitat loss^a,b^
1	0–27	0–12	0–20	≈1	Connected	Weak response	Weak response	Weak response
2	27–50	12–41	20–45	>1	Connected	Accelerating increase	Accelerating decrease	Weak response
3	50–60	41–52	45–55	>1	Fragmented	Decelerating increase	Decelerating decrease	Accelerating increase
4	60–87	52–81	55–85	>1 or <1	Fragmented	Accelerating decrease	Weak response	Accelerating increase
5	87–100	81–100	85–100	>1 or <1	Fragmented	Decelerating decrease	Accelerating decrease, lost dominance	Accelerating increase, gained dominance

^a^According to Fig. 3a. ^b^According to Fig. 3b. ^c^Mean percentages from the preceding two columns rounded to the nearest 5%. ^d^According to Fig. 3c,d. ^e^According to percolation theory and as illustrated in Fig. 1.

### Five phases of habitat loss

The aforementioned four transitions naturally divide a landscape’s degradation process into five phases (Table 1; see Fig. 1 for illustration):

**Phase 1** (occurring at levels of habitat loss from 0% to ca. 20%; Fig. 1a). The landscape is dominated by a single large habitat patch, known from percolation theory as a spanning cluster, which spans across the entire landscape. By far, the most frequent event during this phase is the shrinkage of the spanning patch (Fig. 3a,b). In contrast, patch splitting and patch loss are rare events, as they happen only to the non-spanning smaller patches (Fig. 3a,b). Throughout this phase, all three event probabilities remain almost constant (Fig. 3a,b).

**Phase 2** (occurring at levels of habitat loss from ca. 20% to ca. 45%; Fig. 1b). The spanning patch still exists, but the number and size of the non-spanning smaller patches become significant. Close to the percolation threshold at this phase’s upper boundary, the spanning patch is filamental, which means that the random removal of a single habitat site in the considered lattices of 100 × 100 sites results in its splitting into two habitat patches in about 1/4 of the cases (Fig. 3a,b). In this phase, patch shrinkage and patch splitting co-occur at appreciable frequencies, while patch loss remains rare (Fig. 3a,b). The sensitivities of the frequencies of patch shrinkage and patch splitting to habitat loss (slopes in Fig. 3a,b) both increase toward the percolation threshold, where they reach their peaks.

**Phase 3** (occurring at levels of habitat loss from ca. 45% to ca. 55%; Fig. 1c). This phase is similar to the previous one in terms of the occurrence of the elementary events, while being characterized by a key difference in landscape structure: as habitat loss *q* exceeds the percolation threshold *q*_c_, the landscape is fragmented. In the theoretical limit of infinite landscape size, no spanning patch would exist anymore; however, for finite landscapes, we may still find a spanning patch when *q* − *q*_c_ is small, but the probability of such configurations rapidly declines with an increase of *q* − *q*_c_ and/or of landscape size^21^. This fragmentation implies that it is not possible for a population to spread across the landscape without being obstructed by non-habitat sites, which poses a serious burden on its persistence (see^23^ for a review). In this phase, the frequencies of patch shrinkage and patch splitting continue to decrease and increase, respectively, while the frequency of patch loss also increases, but still remains at low levels (Fig. 3a,b). The sensitivities of the frequencies of patch shrinkage and patch splitting to habitat loss (slopes in Fig. 3a,b) both decrease above the percolation threshold, where they had reached their peaks (Fig. 3a,b).

**Phase 4** (occurring at levels of habitat loss from ca. 55% to ca. 85%; Fig. 1d). In this phase, the frequency of patch shrinkage is surprisingly unresponsive to the further increase of habitat loss, exhibiting a broad plateau (Fig. 3a,b). After having reached its peak at the transition between Phases 3 and 4, the frequency of patch splitting declines with the further increase of habitat loss, and patch loss is gaining importance, gradually becoming as frequent as patch shrinkage (Fig. 3a,b). These effects are caused by decreasing patch sizes.

**Phase 5** (occurring at levels of habitat loss from ca. 85% to 100%; Fig. 1e). In this final phase of landscape degradation, the frequency of patch loss continues to increase super-linearly with further habitat loss, exceeding the frequencies of patch shrinkage and patch splitting, with the latter both continuing to decline with further habitat loss (Fig. 3a,b). Populations inhabiting these kinds of landscapes are confined to live primarily in small habitat patches, which are frequently lost. Therefore, sufficient dispersal between waxing and waning habitat patches is crucial for a population’s persistence.

The aforementioned phases can be observed on a hexagonal lattice (Fig. 3a) and on a square lattice with von Neumann neighbourhood (Fig. 3b). In the hexagonal lattice, each phase starts later, i.e., at a higher level of habitat loss (Table 1). The reason for this is the higher number of connections per site (degree): each site is connected to six others, as opposed to four others in the square lattice with von Neumann neighbourhood. Therefore, more sites need to be removed from a hexagonal lattice for causing a habitat patch to split, or for isolating a habitat site completely, making it less vulnerable to patch loss.

### Effective habitat loss

To assess the severity of impact of landscape degradation on a population, we assume that sustaining a viable local population requires a habitat patch with a minimum of *m* habitat sites. When a single isolated habitat site is sufficient for sustaining a local population (*m* = 1), then every site removal decreases the population’s living area by exactly one site. In contrast, when *m* = 2, then fragmentation of a habitat patch of size 3 creates two fragments, each of size 1, which effectively removes three habitat sites from the population’s living area. In this manner, every elementary local event is evaluated according to the effective habitat loss it causes. Some site removals cause no loss of habitat, as they affect patches that are already too small to sustain a local population. These habitat patches are part of the landscape; they are just lost from the population’s living area.

For any given total level of habitat loss *q*, we determine the amplification factor of effective habitat loss by averaging the effective habitat loss (i.e., the loss of a population’s living area) over a large number of elementary events (6 · 10^6^), each involving an actual habitat loss of one habitat site. This factor starts to exceed 1 appreciably when the level of habitat loss *q* exceeds roughly 30% (Fig. 3c,d), which is well below the percolation threshold and roughly coincides with the transition from Phase 1 to Phase 2. The amplification factor of effective habitat loss peaks at levels of habitat loss well above the percolation threshold, close to the level of habitat loss at which the probability of patch splitting is maximal (compare Fig. 3a–d). This peak shifts toward lower levels of habitat loss *q* as the minimal patch area needed for a viable local population increases (Fig. 3c,d). After reaching its peak, the amplification factor of effective habitat loss strongly declines and typically drops below 1 in Phase 4 (Fig. 3c,d), which means that by removing a single habitat site on average less than one habitat site is lost from the population’s living area. This is because in this phase most habitat patches are already too small for sustaining a local population, so that the further removal of a habitat site often causes no further loss from a population’s living area. Naturally, this effect is stronger and starts earlier, i.e., at lower levels of habitat loss *q*, for populations requiring habitat patches of larger minimal size *m*.

## Discussion

Here we have presented a dynamical view of habitat loss by observing the probabilities of elementary local events on the spatial mesoscale of individual habitat patches. Our framework for studying changing landscapes allows us to identify three additional landscape transitions beside the one associated with the well-known percolation threshold. The four corresponding thresholds demarcate five distinct phases of habitat loss. Phase 1 begins from a pristine habitat and is characterized by the shrinkage of contiguous habitat area. In Phase 2, detached habitat patches begin to appear, even though a spanning patch still exists. The percolation threshold marks the transition to Phase 3, in which the effective habitat loss peaks, and at the end of which the frequency of patch splitting reaches its peak. It is important to emphasize that – although the connectivity of habitat sites plummets at the percolation threshold – our results demonstrate that the severity and frequency of patch splitting peaks at higher levels of habitat loss. It is in Phase 3 that organisms requiring a larger number of habitat sites for sustaining a viable local population experience a peak in effective habitat loss. Phase 4 commences with the peak in the probability of patch splitting and is characterized by a nearly constant probability of patch shrinkage. Finally, in Phase 5, habitat loss is so severe that the landscape mainly consists of isolated habitat sites, which then disappear one-by-one as habitat cover decreases.

The same process can alternatively be described from the opposite direction, which is a trend toward habitat gain. For example, when sites with arable land are created, the corresponding habitat patches may become suitable for weeds or other agricultural pests, increasing their population sizes, as well as their probabilities of spreading across the landscape.

Dynamical Neutral Landscape Models (DNLMs) serve as natural benchmarks and references to which data observed on real landscapes can be compared. High correlations between a landscape index and a response variable measured on artificial landscapes have often been confirmed by measurements on real landscapes^16^. Real landscapes differ from percolation maps in at least two important characteristics. (1) Non-habitat patches can be traversed up to some distance^85–87^, and thus, habitat patches can still be functionally connected^14^ in spite of their physical fragmentation. (2) Real landscapes are, in general, not as randomly structured as percolation maps, but instead exhibit some degree of aggregation^34,88–90^. When habitat sites are aggregated, patch splitting is less likely, and thus occurs at higher levels of habitat loss. This is corroborated, for example, by observations on birds and mammals^85^, which have led to the conclusion that a landscape becomes significantly fragmented for these species only when 60–70% of the habitat is destroyed. Habitat fragmentation on a percolation map can thus be considered as a worst-case scenario.

Our numerical investigations in the present study have been carried out on a percolation map, since this is the first^20^ and best-studied NLM, and also the most widely used in spatially explicit models^13,15,30,67,68,91–94^. Percolation maps are often applied to study habitat fragmentation because of the critical behaviour they exhibit^13,20–22^. In contrast, habitat change in other models of spatial ecology has mostly been defined in terms of disturbance regimes^13,15,91,95–100^. Up to now, NLMs with temporal changes have scarcely been considered (for exceptions, see^23,24,48,56,69,73^). The DNLM framework could be extended in a variety of ways. First, the dynamical properties of NLMs other than percolation maps could be similarly investigated. This perspective could also be applied to landscape models defined in continuous space (rather than on a lattice, as in our present study): In those contexts, it would then be interesting to examine how the dynamical landscape properties are changing when scanning through ranges of distances for defining whether nearby locations are regarded as being connected or not. The frequency distribution of events on other landscapes is expected to be different. In particular, on landscapes with spatially aggregated patterns of habitat sites, we expect patch splitting to dominate at higher levels of habitat loss. Second, the dynamics of populations can be described in greater mechanistic detail. For example, the costly – but in general not impossible – movement of individuals through non-habitat sites could be included (for an example of such a model, see^57^). The explicit study of population dynamics within the patches, including the study of extinction times, is also a promising direction for future research (see^23^ about connecting habitat dynamics and population dynamics in a percolation-theoretical framework). Third, in our present study, we have removed habitat sites randomly, according to a spatially uniform probability distribution. Future research could consider more elaborate models of habitat loss, reflecting real-word processes such as agricultural land-use expansion or other anthropogenic environmental changes. In this context, an examination of contagious habitat loss^101^ would be of particular interest.

The importance of the percolation threshold for understanding landscape dynamics and habitat fragmentation has been recognized in the literature from the early days of landscape ecology^20^ (see reviews of relevant studies in^58,102^). Here we have suggested considering three additional thresholds that are fundamentally related to understanding the dynamics of habitat patches and the impacts of habitat loss on different organisms that require habitat patches of a certain minimal size for their local survival. Altogether, the four thresholds we have defined, examined, and discussed here distinguish five characteristic phases of habitat loss. We believe that the distinction between these phases according to a landscape’s dynamical properties, focussing on the patch-scale events, is crucial for the protection of species subject to habitat loss and fragmentation. In particular, important qualitative changes in the dynamics of habitat patches can be revealed by studying the frequencies of the elementary local events we have investigated here. Habitat changes thus becoming detectable will often foreshadow critical landscape transitions to occur, which allows scientists and managers to take action before disastrous and irreversible damage is done.

## Data Availability

The datasets generated and analysed for the current study are available from the corresponding author on reasonable request.
